# Predictors of 30‐Day Recurrent Emergency Department Visits for Hyperglycemia in Patients With Diabetes: A Multicentre Prospective Cohort Study

**DOI:** 10.1111/acem.70133

**Published:** 2025-08-27

**Authors:** Justin W. Yan, Nicolas Woods, Kristine Van Aarsen, Joe Thorne, Igor Karp, Tamara Spaic, Selina L. Liu, Ian G. Stiell, For the Network of Canadian Emergency Researchers

**Affiliations:** ^1^ Division of Emergency Medicine, Department of Medicine, Schulich School of Medicine and Dentistry Western University London Ontario Canada; ^2^ Lawson Health Research Institute London Ontario Canada; ^3^ Department of Epidemiology and Biostatistics, Schulich School of Medicine and Dentistry Western University London Ontario Canada; ^4^ Division of Endocrinology and Metabolism, Department of Medicine, Schulich School of Medicine and Dentistry Western University London Ontario Canada; ^5^ Department of Emergency Medicine University of Ottawa Ottawa Ontario Canada; ^6^ Ottawa Hospital Research Institute Ottawa Ontario Canada

**Keywords:** diabetes, emergency department, hyperglycemia, patient outcomes, predictors/risk factors, recurrent visits

## Abstract

**Objectives:**

Identifying predictors of increased healthcare utilization for hyperglycemia may have important implications for designing interventions to improve patient outcomes and reduce costs. Studies examining predictors of 30‐day recurrent ED hyperglycemia visits have been limited due to their retrospective nature. This study's objective was to prospectively identify predictors of 30‐day recurrent ED visits for hyperglycemia in patients with diabetes.

**Methods:**

We conducted a multicentre, prospective cohort study of adults ≥ 18 years at one of four Canadian tertiary care, academic EDs with a diagnosis of hyperglycemia, diabetic ketoacidosis, or hyperosmolar hyperglycemic state. Multivariable logistic regression analysis was used to identify variables independently associated with recurrent 30‐day ED visits for hyperglycemia.

**Results:**

We enrolled 594 patients; 80 (13.5%) had a recurrent ED visit for hyperglycemia within 30 days. Independently associated predictors of 30‐day recurrent visits on complete case analysis include substance abuse history (odds ratio [OR] 2.32, 95% confidence interval [CI]: 1.23–4.38) and initial laboratory blood glucose (OR 1.04, 95% CI: 1.01–1.07), while a new diabetes diagnosis was negatively associated (OR 0.29, 95% CI: 0.09–0.94). Sensitivity analysis using multiple imputation for missing data found the following independently associated variables: substance abuse history (OR 2.55, 95% CI: 1.34–4.85), previous ED visit within the past 14 days (OR 2.14, 95% CI: 1.02–4.48), and initial laboratory blood glucose (OR 1.04, 95% CI: 1.01–1.07). Two variables were negatively associated: recent hospitalization within the past 30 days (OR 0.40, 95% CI: 0.19–0.98) and new diabetes diagnosis (OR 0.37, 95% CI: 0.14–0.97).

**Conclusions:**

This multicentre prospective study reports predictors independently associated with 30‐day recurrent ED visits for hyperglycemia. These predictors should be considered by ED clinicians when making disposition and follow‐up plans for this important patient population, and future interventions should explore the interaction between hyperglycemia and substance use to prevent recurrent ED visits and reduce healthcare system costs and utilization.

## 1 Introduction

Diabetes mellitus has been described as “the epidemic of the century” globally [1], and studies have demonstrated that emergency department (ED) visits and hospital admissions for hyperglycemic crises are only increasing [2, 3]. Identifying predictors of increased healthcare resource utilization for hyperglycemia may have important implications for designing interventions to improve patient outcomes and reduce system costs [4]. While multiple studies have examined predictors of 30‐day recurrent ED visits for hyperglycemia in patients with diabetes, conclusions have been limited by their retrospective nature and prospective data collection is lacking [5, 6, 7]. Therefore, the objective of this study was to prospectively identify predictors of 30‐day recurrent visits in patients presenting to the ED for hyperglycemia.

## 2 Methods

### 2.1 Study Design and Setting

We conducted a multicentre prospective cohort study of eligible adult patients with an ED diagnosis of hyperglycemia, diabetic ketoacidosis, or hyperosmolar hyperglycemic state. The setting was the EDs of two urban, tertiary care, academic centres comprising four sites (London Health Sciences Centre Victoria and University Campuses, The Ottawa Hospital Civic and General Campuses) in Ontario, Canada, with a combined census of over 300,000 ED visits annually. Enrolment occurred from 2017 to 2020 at the two London sites until all research activities paused temporarily due to the COVID‐19 pandemic in March 2020; however, they resumed in 2022. The two Ottawa sites enrolled patients from December 2022 to November 2023 until the target sample size was achieved. The study protocol was approved by Western University's Health Sciences Research Ethics Board and the Ottawa Health Science Network Research Ethics Board and was conducted in accordance with the STROBE guidelines for reporting observational studies (Appendix S1) [8].

### 2.2 Study Population

All adult (≥ 18 years) ED patients with a final ED diagnosis of hyperglycemia (> 11.0 mmol/L), diabetic ketoacidosis, or hyperosmolar hyperglycemic state as determined by the treating physician were eligible to be enrolled. This included patients with both type 1 and type 2 diabetes, those who were both admitted and discharged, and patients with co‐morbid diagnoses in addition to hyperglycemia (e.g., infection, cardiac ischemia, etc.). We excluded patients who were initially assessed at a peripheral or community hospital and were transferred to our study sites for ongoing management by an inpatient service; those who were unable (and absence of a substitute decision‐maker) to provide informed consent; and those who had previously been enrolled in our study on a prior ED visit for hyperglycemia.

### 2.3 Outcomes

The primary outcome was an unplanned return ED visit for hyperglycemia within 30 days of initial presentation. Secondary outcomes included unplanned admission to hospital or intensive care unit for hyperglycemia, or death within 30 days of the index ED visit.

### 2.4 Informed Consent Process

Informed consent was required for patients to be contacted for telephone follow‐up at all sites. At London Health Sciences Centre, potentially eligible patients were identified and reviewed from the local site's ED tracking system by a trained research assistant, and if eligible, were flagged for a member of the circle of care to obtain written informed consent. Emergency physicians confirmed eligibility for enrolment and obtained informed consent for the research team to contact the patient for telephone follow‐up at 14 and 30‐day post‐ED visit. At The Ottawa Hospital, research assistants screened the daily ED visit log for eligible participants, and those who had previously indicated they were willing to participate in research (according to their electronic medical record) were contacted for consent and follow‐up. The age and sex of missed eligible patients and those who declined to participate were compared to the enrolled participants to assess for selection bias.

### 2.5 Data Collection

For each enrolled patient, study data pertaining to their ED visit was abstracted from the local site's electronic medical record into a study‐specific REDCap case record form, which consisted of six data instruments for the health records data abstraction (demographic information, medical history, visit history, ED presentation variables including most likely precipitant of hyperglycemia as determined or documented by the treating physician, diagnostic and investigation variables, and discharge and disposition data) and two data instruments for the prospective telephone follow‐up (14‐ and 30‐day telephone follow‐up variables). Research assistants contacted patients at 14 and 30 days via telephone to obtain follow‐up data regarding clinical outcomes, including repeat visits to see a healthcare provider outside our study sites, changes in diabetic medications, and time taken off work or school. Further demographic data including ethnicity, socioeconomic status, and education level were also asked during the telephone follow‐up. Patients unable to be reached after three telephone attempts over three different days were declared lost to follow‐up. Research assistants also performed a review of patients' medical records at 30 days from their initial ED visit to determine if they had any subsequent hyperglycemia‐related ED visits, hospital or intensive care unit admissions, or died.

### 2.6 Statistical Analysis

Relevant patient characteristics were summarized using descriptive statistics, and differences between groups were assessed using chi‐squared, Fisher's exact test, and *t*‐test as appropriate. Patient characteristics that were thought to be related to the outcomes of interest were selected based on expert opinion, prior research about the epidemiology of the disease process, and hypothesized relationships between potential independent variables and recurrent hyperglycemia visits. Based on these variables, a multivariable logistic regression model with bootstrapped standard errors was fitted, predicting the odds of an unplanned return hyperglycemia visit within 30 days. Listwise deletion was used for the primary regression model; though, due to missing data for some variables, multiple imputation by chained equations was used to impute for these, with a subsequent multivariable regression being run as a sensitivity analysis to ensure no bias was introduced due to listwise deletion.

After fitting the listwise deletion multivariable model, the predicted probabilities were used to generate a receiver operating characteristic (ROC) curve using the outcome variable and the predicted probabilities variable; the area under the ROC curve for the model was determined. To quantify the model's goodness‐of‐fit, we computed the Hosmer–Lemeshow statistic [9], and the discriminatory ability was assessed by the Brier score [10], which ranges from 0 (perfect accuracy) to 1 (perfect inaccuracy), with lower scores representing a more accurate model.

To estimate the sample size required for the multivariable regression model, the formula by Peduzzi et al. was used, *N* = 10 *k*/*p*; where *p* is the estimated proportion of patients who will have an unplanned recurrent ED visit for hyperglycemia within 30 days and *k* is the number of covariates (independent variables) to be included in the model [11]. It was expected that up to 7 covariates would be included in the final multivariable model and estimated that 15% of patients would have the outcome at 30 days, resulting in an estimated sample size of 467 patients. This sample size was increased by 20% to account for potential loss to follow‐up, resulting in a final sample size of 560 patients. All statistical analyses were completed using Stata version 18.0 (Statacorp LLC, College Station, TX).

## 3 Results

During the study period, 594 patients were enrolled; 80 (13.5%) had an unplanned 30‐day return ED visit for hyperglycemia, while 514 did not. Individual patient characteristics are summarized in Table 1, separated by who had and did not have the primary outcome. In the cohort, 53.4% were male, with a mean (SD) age of 52.1 (18.2) years. Over half the patients (57.6%) had type 2 diabetes, and 24.9% had type 1 diabetes; 57.8% had a family physician, and 29.6% were followed by an endocrinologist. The most common comorbidities were hypertension (49.7%), dyslipidemia (32.3%), and psychiatric illness (26.4%). We were able to reach 367 patients for telephone follow‐up, providing education, race/ethnicity, and annual household income data on 61.8% of our patients, which are also summarized in Table 1.

**TABLE 1:** Characteristics of 594 included hyperglycemia patients organized by whether they had a recurrent visit to the ED within 30 days for hyperglycemia (*n* = 80) or not (*n* = 514).

	Total unique patients (*n* = 594)	No recurrent visit (*n* = 514)	Recurrent visit for hyperglycemia (*n* = 80)	*p*
*Patient Characteristic*				
Sex, *n* (%)				
Female	275 (46.3%)	244 (47.5%)	31 (38.8%)	0.09
Male	317 (53.4%)	269 (52.3%)	48 (60.0%)	
Intersex	2 (0.4%)	1 (0.2%)	1 (1.2%)	
Age, years (mean ± SD)	52.1 ± 18.2	51.7 ± 18.3	54.2 ± 17.6	0.13
Long‐term care, *n* (%)	16 (2.7%)	12 (2.3%)	4 (5.0%)	0.25
No fixed address, *n* (%)	10 (1.7%)	6 (1.2%)	4 (5.0%)	0.03*
Arrival mode, *n* (%)				
Self	406 (68.4%)	362 (70.4%)	44 (55.0%)	0.03*
EMS	187 (31.5%)	152 (29.6%)	35 (43.8%)	
Police	1 (0.2%)	0 (0.0%)	1 (1.2%)	
Study site, *n* (%)				
LHSC—Victoria Campus	264 (44.4%)	223 (43.4%)	41 (51.3%)	0.22
LHSC—University Campus	179 (30.1%)	150 (29.2%)	29 (36.3%)	
TOH—General Campus	81 (13.6%)	76 (14.8%)	5 (6.3%)	
TOH—Civic Campus	70 (11.8%)	65 (12.7%)	5 (6.3%)	
Previously known history of diabetes, *n* (%)				
New diagnosis/no history of diabetes	101 (17.0%)	96 (18.7%)	5 (6.2%)	0.02*
Type 1	148 (24.9%)	124 (24.1%)	24 (30.0%)	
Type 2	342 (57.6%)	291 (56.6%)	51 (63.8%)	
Gestational	3 (0.5%)	3 (0.6%)	0 (0.0%)	
Diabetes medications, *n* (%)				
Insulin	302 (50.8%)	252 (49.0%)	50 (62.5%)	0.02*
Oral hypoglycemic	273 (46.0%)	230 (44.7%)	43 (53.8%)	0.13
Not on diabetes medications prior to visit	138 (23.2%)	131 (25.5%)	7 (8.8%)	< 0.01*
Followed by, *n* (%)				
Family physician	343 (57.8%)	285 (55.4%)	58 (72.5%)	< 0.01*
Endocrinology	176 (29.6%)	152 (29.6%)	24 (30.0%)	0.94
Diabetes education nurse	59 (9.9%)	49 (9.5%)	10 (12.5%)	0.41
Internal medicine	53 (8.9%)	49 (9.5%)	4 (5.0%)	0.19
Unknown/Not documented	166 (27.9%)	152 (29.6%)	14 (17.5%)	0.02*
Comorbidities, *n* (%)				
Hypertension	295 (49.7%)	254 (49.4%)	41 (51.2%)	0.76
Hyperlipidemia	192 (32.3%)	166 (32.3%)	26 (32.5%)	0.97
Psychiatric illness	157 (26.4%)	126 (24.5%)	31 (38.8%)	< 0.01*
Coronary artery disease	90 (15.2%)	74 (14.4%)	16 (20.0%)	0.19
Substance Abuse	81 (13.6%)	59 (11.5%)	22 (27.5%)	< 0.01*
Chronic obstructive pulmonary disease/asthma	75 (12.6%)	66 (12.8%)	9 (11.2%)	0.69
Cancer	68 (11.4%)	64 (12.5%)	4 (5.0%)	0.05
Stroke/Transient ischemic attack	57 (9.6%)	44 (8.6%)	13 (16.2%)	0.03*
Chronic renal failure	28 (4.7%)	26 (5.1%)	2 (2.5%)	0.41
Peripheral vascular disease	17 (2.9%)	12 (2.3%)	5 (6.2%)	0.06
Dementia	13 (2.2%)	10 (1.9%)	3 (3.8%)	0.40
None of the above	162 (27.3%)	147 (28.6%)	15 (18.8%)	0.07
*Visit Characteristic on Arrival*				
CTAS, *n* (%)	*n* = 593	*n* = 513	*n* = 80	0.11
1	10 (1.7%)	8 (1.6%)	2 (2.5%)	
2	307 (51.8%)	257 (50.1%)	50 (62.5%)	
3	258 (43.5%)	233 (45.4%)	25 (31.2%)	
4	17 (2.9%)	14 (2.7%)	3 (3.8%)	
5	1 (0.2%)	1 (1.9%)	0 (0.0%)	
EMS blood glucose, mmol/L (mean ± SD)	*n* = 75	*n* = 62	*n* = 13	0.22
	23.9 ± 7.5	23.6 ± 7.7	25.4 ± 6.3	
Temperature, °C (mean ± SD)	*n* = 591	*n* = 511	*n* = 80	0.21
	36.6 ± 0.6	36.6 ± 0.6	36.7 ± 0.5	
Systolic blood pressure, mmHg (mean ± SD)	140.3 ± 23.8	140.1 ± 23.5	141.0 ± 25.4	0.38
Diastolic blood pressure, mmHg (mean ± SD)	83.0 ± 15.0	83.1 ± 14.8	82.7 ± 16.0	0.41
Heart rate, beats per minute (mean ± SD)	94.0 ± 18.8	94.0 ± 18.9	94.2 ± 18.3	0.46
Respiratory rate, breaths per minute (mean ± SD)	*n* = 593	*n* = 513	*n* = 80	0.39
	18.5 ± 3.8	18.5 ± 3.8	18.4 ± 3.8	
Oxygen saturation (mean ± SD)	*n* = 592	*n* = 513	*n* = 79	0.40
	97.3 ± 2.1	97.3 ± 2.1	97.4 ± 1.9	
Initial laboratory blood glucose, mmol/L (mean ± SD)	*n* = 576	*n* = 499	*n* = 77	< 0.01*
	23.8 ± 9.7	23.4 ± 9.6	26.8 ± 10.0	
On oxygen at arrival, *n* (%)	*n* = 592	*n* = 513	*n* = 79	0.55
	24 (4.1%)	20 (3.9%)	4 (5.1%)	
Final hyperglycemic diagnosis, *n* (%)				
Hyperglycemia/Diabetes mellitus	456 (76.8%)	394 (76.7%)	62 (77.5%)	0.88
Diabetes ketoacidosis	115 (19.4%)	100 (19.5%)	15 (18.8%)	
Hyperosmolar hyperglycemic state	23 (3.9%)	20 (3.9%)	3 (3.8%)	
Previous 14 day ED visit, *n* (%)	53 (8.9%)	39 (7.6%)	14 (17.5%)	< 0.01*
*Telephone Follow‐up Characteristic*				
Highest level of education completed, *n* (%)	*n* = 348	*n* = 308	*n* = 40	0.58
No schooling	3 (0.9%)	2 (0.6%)	1 (2.5%)	
Grade 8/Elementary	49 (14.1%)	43 (14.0%)	6 (15.0%)	
Secondary school	121 (34.8%)	103 (33.4%)	17 (42.5%)	
College diploma	109 (31.3%)	98 (31.8%)	11 (27.5%)	
Bachelor's degree	48 (13.8%)	44 (14.3%)	4 (10.0%)	
Master's, PhD, or professional	18 (5.2%)	17 (5.5%)	1 (2.5%)	
Ethnic minority or racial heritage, *n* (%)	*n* = 339	*n* = 301	*n* = 38	0.19
Native American/Indigenous	24 (7.1%)	20 (6.6%)	4 (10.5%)	
White or Caucasian	251 (74.0%)	222 (73.6%)	29 (76.3%)	
Hispanic or Latino	3 (0.9%)	3 (1.0%)	0 (0.0%)	
Black or African Canadian	15 (4.4%)	11 (3.7%)	4 (10.5%)	
Middle Eastern/Arab	15 (4.4%)	15 (5.0%)	0 (0.0%)	
East Asian/Pacific Islander	2 (0.6%)	2 (0.7%)	0 (0.0%)	
South Asian (e.g., Indian, Pakistani)	8 (2.4%)	7 (2.3%)	1 (2.6%)	
Other	21 (6.2%)	21 (7.0%)	0 (0.0%)	
Approximate household annual income, *n* (%)	*n* = 275	*n* = 242	*n* = 33	
Less than $25,000	87 (31.6%)	72 (29.8%)	15 (45.6%)	
$25,000–$49,999	76 (27.6%)	72 (29.8%)	4 (12.1%)	
$50,000–$74,999	48 (17.5%)	39 (16.1%)	9 (27.3%)	0.01*
$75,000–$99,999	24 (8.7%)	20 (8.3%)	4 (12.1%)	
$100,000 or more	40 (14.5%)	39 (16.1%)	1 (3.0%)	

Abbreviations: CTAS = Canadian Triage and Acuity Scale, EMS = emergency medical services, LHSC = London Health Sciences Centre, SD = standard deviation, TOH = The Ottawa Hospital.

* Statistically significant at the *α* = 0.05 level, as determined via chi‐square, Fisher's exact test, or *t*‐test.

The most common precipitants for hyperglycemia were medication non‐adherence (19.9%), pre‐existing poor glycemic control (12.8%), new‐onset diabetes (12.6%), followed by infection‐induced hyperglycemia (8.6%) (Table 2). Table 2 also summarizes the results of bloodwork and urinalysis performed and interventions including intravenous fluids, insulin administration, and critical care interventions, such as inotropes and airway management.

**TABLE 2:** Likely precipitant, investigations, and interventions on 594 emergency department patients presenting for hyperglycemia.

Likely precipitant (%)	*N* = 594
Non‐adherence	118 (19.9)
Poor glycemic control	76 (12.8)
New diagnosis of diabetes	75 (12.6)
Infection‐induced	51 (8.6)
Respiratory	20 (39.2)
Gastrointestinal	13 (25.5)
Urinary	11 (21.6)
Skin/Soft tissue	3 (5.9)
Genital/Gynecologic	2 (3.9)
Alcohol‐related	8 (1.4)
Cardiac ischemia	4 (0.7)
Other	96 (16.2)
No specific precipitant	210 (35.4)
Bloodwork completed, mean (SD)	
No, *n* (%)	10 (1.7)
Yes, *n* (%)	584 (98.3)
Hemoglobin, g/L	137.4 (21.7)
Leukocytes, ×10^9^/L	10.2 (4.5)
Sodium, mmol/L	132.9 (5.1)
Potassium, mmol/L	4.5 (2.0)
Chloride, mmol/L	94.2 (6.2)
CO_2_, mmol/L	21.7 (6.3)
Anion Gap, mmol/L	17.2 (7.4)
Glucose, mmol/L	23.8 (9.7)
Urea, mmol/L	8.6 (6.2)
Creatinine, μmol/L	98.5 (88.3)
Lactate, mmol/L	2.3 (1.5)
Serum ketones/beta‐hydroxybutyrate, *n* (%)	242 (40.7)
Blood gas pH	7.3 (0.1)
Blood gas pCO_2_, mmHg	43.1 (10.2)
Blood gas pO_2_, mmHg	37.2 (15.0)
Blood gas HCO_3_, mmol/L	24.6, (7.3)
Urinalysis completed	*n* (%)
No	326 (54.9)
Yes	268 (45.1)
Glucose detected	257 (95.9)
Ketones detected	163 (60.8)
Blood detected	140 (52.2)
Proteins detected	111 (41.9)
Leukocytes detected	44 (16.4)
Nitrites detected	15 (5.6)
Interventions	*n* (%)
No	105 (17.7)
Yes	489 (82.3)
IV fluid infusion	277 (46.6)
IV fluid bolus	172 (29.0)
Insulin bolus	187 (31.5)
Insulin infusion	129 (21.7)
Oral hypoglycemic	29 (4.9)
IV Sodium bicarbonate	18 (3.0)
Airway intervention	2 (0.3)
Inotrope	1 (0.2)
Other	170 (28.6)

Abbreviation: IV = intravenous.

Table 3 presents the in‐ED and 30‐day outcomes for the included patients. The final hyperglycemic diagnosis was “hyperglycemia/diabetes” in 72.1%, “diabetic ketoacidosis” in 19.4%, and hyperosmolar hyperglycemic state in 3.9% of patients. Over two‐thirds of patients (66.8%) were discharged from the ED, and 32.5% were admitted for a mean (SD) length of stay of 5.4 (8.4) days; 94.8% of admissions were to a medical ward and 5.2% to the intensive care unit. At 30 days, 80 (13.5%) had a return ED visit for hyperglycemia, 38 (6.4%) were hospitalized for hyperglycemia, and 4 (0.7%) died.

**TABLE 3:** Final diagnoses, consultations, disposition, and outcomes for 594 emergency department hyperglycemia patients.

Outcome	*n* (%)
Final hyperglycemic diagnosis	
Hyperglycemia or diabetes	456 (76.8)
Diabetic ketoacidosis	115 (19.4)
Hyperosmolar hyperglycemic state	23 (3.9)
Consultations in ED	
Internal medicine	171 (28.8)
Endocrinology	39 (6.6)
Intensive care unit	6 (1.0)
Other (general surgery, cardiology, urology, respirology, etc.)	75 (12.6)
No documented consultation	373 (62.8)
Disposition from ED	
Discharged	397 (66.8)
Admitted	193 (32.5)
To ward	183 (94.8)
To intensive care unit	10 (5.2)
Mean length of stay (SD)	5.4 (8.4)
Left against medical advice	4 (0.7)
Death in ED	0 (0.0)
Death in hospital after admission	3 (0.5)
30‐day outcomes	
Return visit to ED for hyperglycemia	80 (13.5)
Hospital admission for hyperglycemia	38 (6.4)
ICU admission for hyperglycemia	0 (0.0)
Death	4 (0.7)
None of the above	498 (83.8)

Abbreviations: ED = emergency department; ICU = intensive care unit.

**FIGURE 1:**
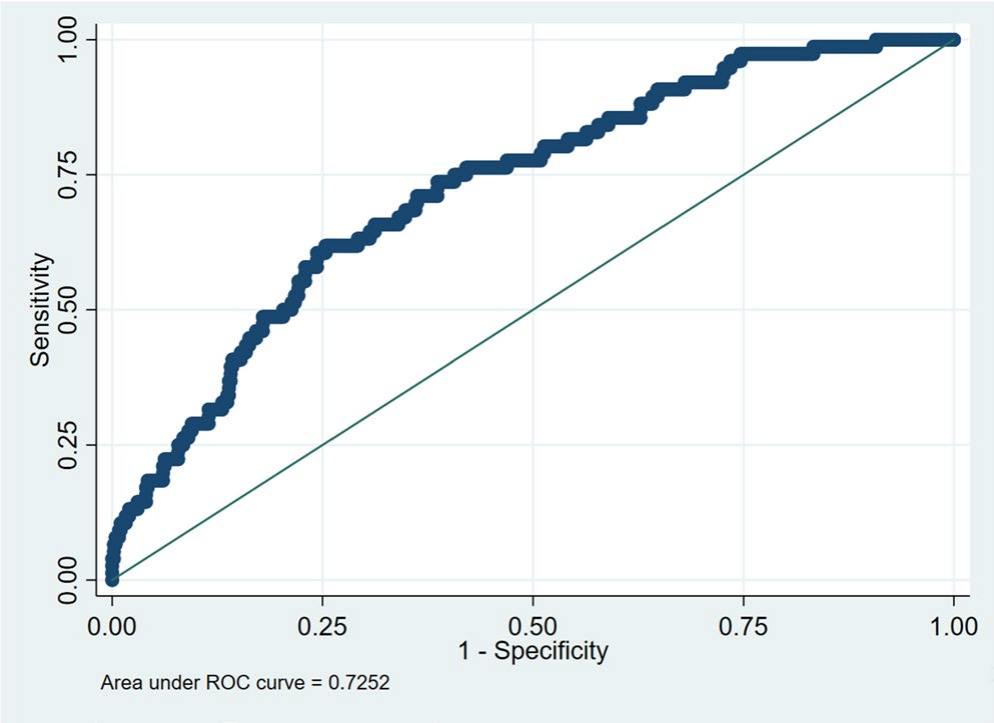
Receiver operating curve for multivariable regression model.

Of the 594 total patients, 21 had a high number of missing values and were excluded, so we conducted a complete case multivariable logistic regression analysis on 573 patients with 5000 bootstrapping samples. Factors independently associated with an unplanned 30‐day repeat ED visit for hyperglycemia include: history of substance abuse, defined as having either opioid and/or substance (illicit drug) use disorder (odds ratio [OR] 2.32, 95% confidence interval [CI] 1.23–4.38), initial laboratory blood glucose level (OR 1.04, 95% CI: 1.01–1.07), while a new diagnosis of diabetes (i.e., first‐time presentation for hyperglycemia with no documented history of having diabetes) was negatively associated with the primary outcome (OR 0.29, 95% CI: 0.09–0.94) (Table 4). We explored various dichotomous cutpoints for initial blood glucose level (e.g., > 15, > 18, > 20, and > 25 mmol/L) in order to make this variable more useful for clinicians, but the results were not impacted by this exploration. The Hosmer–Lemeshow statistic was 0.56, demonstrating that the model adequately fits the data. The Brier score, which assesses the model's calibration and discriminatory ability, was 0.10. The ROC curve for the multivariable regression model is presented in Figure 1, and the area under the curve for the model was 0.73.

**TABLE 4:** Complete case analysis using 5000 bootstrapped samples: Variables independently associated with unplanned recurrent ED visits for hyperglycemia within 30 days (*n* = 573).

Variable	Bootstrap‐adjusted odds ratio (95% CI)	Bootstrap‐adjusted standard error	*p*
Age, years	1.00 (0.99–1.02)	0.01	0.77
Sex			
Female	Reference		
Male	1.62 (0.94–2.81)	0.45	0.08
Final hyperglycemic diagnosis			
Hyperglycemia/Diabetes mellitus	Reference		
Diabetic ketoacidosis	1.21 (0.45–3.25)	0.61	0.71
Hyperosmolar hyperglycemic state	0.70 (0.16–3.03)	0.52	0.63
ED disposition			
Discharged/Left against medical advice	Reference		
Hospitalized	0.48 (0.21–1.06)	0.20	0.07
**Initial laboratory blood glucose**	**1.04** (**1.01–1.07**)	**0.02**	**0.03** *
Arrival by EMS	1.48 (0.82–2.68)	0.45	0.19
CTAS			
1 or 2	Reference		
3, 4 or 5	0.60 (0.33–1.11)	0.19	0.11
Diabetes status			
Pre‐existing diabetes diagnosis	Reference		
**New diabetes diagnosis/no diabetes history**	**0.29** (**0.09–0.94**)	**0.17**	**0.04** *
Followed by family physician	1.74 (0.94–3.21)	0.55	0.08
**History of substance use**	**2.32** (**1.23–4.38**)	**0.75**	**< 0.01** *
Previous ED visit in past 14 days	1.88 (0.83–4.28)	0.79	0.13

Abbreviations: CTAS = Canadian Triage and Acuity Scale, ED = emergency department, EMS = emergency medical services.

* Statistically significant at the *α* = 0.05 level.

As a sensitivity analysis, we performed a multivariable regression analysis with multiple imputation for missing data for all 594 patients and present this model in Table 5. In this analysis, variables independently associated with a 30‐day recurrent unplanned ED visit for hyperglycemia include a history of substance abuse (OR 2.55, 95% CI: 1.34–4.85), a previous visit to the ED within the past 14 days (OR 2.14, 95% CI: 1.02–4.48), and the initial laboratory blood glucose level (OR 1.04, 95% CI: 1.01–1.07). Two variables were found to be negatively associated with the primary outcome: recent hospitalization within the past 30 days (OR 0.40, 95% CI: 0.19–0.98) and a new diabetes diagnosis (OR 0.37, 95% CI: 0.14–0.97).

**TABLE 5:** Sensitivity analysis with multiple imputation for all cases: Variables independently associated with unplanned recurrent ED visits for hyperglycemia within 30 days (*n* = 594).

Variable	Odds ratio (95% CI)	Standard error	*p*
Age	1.00 (0.98–1.02)	0.01	0.99
Sex			
Female	Reference		
Male	1.58 (0.92–2.72)	0.44	0.10
Final hyperglycemic diagnosis			
Hyperglycemia/Diabetes mellitus	Reference		
Diabetic ketoacidosis	1.20 (0.46–3.10)	0.58	0.71
Hyperosmolar hyperglycemic state	0.73 (0.16–3.43)	0.58	0.69
ED disposition			
Discharged/Left against medical advice	Reference		
**Hospitalized**	**0.40** (**0.19–0.98**)	**0.18**	**0.05** *
**Initial laboratory blood glucose**	**1.04** (**1.01–1.07**)	**0.02**	**0.02** *
Arrival by EMS	1.68 (0.95–2.99)	0.49	0.08
CTAS			
1 or 2	Reference		
3, 4 or 5	0.66 (0.38–1.15)	0.19	0.15
Diabetes status			
Pre‐existing diabetes diagnosis	Reference		
**New diabetes diagnosis/no diabetes history**	**0.37** (**0.14–0.97**)	**0.18**	**0.04** *
Followed by family physician	1.73 (0.96–3.11)	0.52	0.07
**History of substance use**	**2.55** (**1.34–4.85**)	**0.84**	**< 0.01** *
**Previous ED visit in past 14 days**	**2.14** (**1.02–4.48**)	**0.81**	**0.04** *
Race/Ethnicity			
White or Caucasian	Reference		
Visible minority	1.22 (0.50–3.00)	0.56	0.66
Annual household income	0.96 (0.71–1.31)	0.15	0.80

Abbreviations: CTAS = Canadian Triage and Acuity Scale, ED = emergency department, EMS = emergency medical services.

* Statistically significant at the *α* = 0.05 level.

After study closure, we examined the characteristics of patients who either declined enrolment and/or contact for research purposes and those who were missed in order to check for sampling bias. The mean (SD) age of those potentially eligible patients who were not included was 54.9 (19.7) years, and 53.9% were male, similar to our cohort.

## 4 Discussion

### 4.1 Interpretation

This multicentre, prospective cohort study examined predictors of recurrent unplanned visits to the ED for hyperglycemia within 30 days. In both our complete case and sensitivity analyses, the strongest risk factor for recurrent visits to the ED for hyperglycemia in 30 days was a history of substance abuse. Additional variables independently associated with recurrent ED visits for hyperglycemia within 30 days included initial laboratory blood glucose level and recent ED visit within the past 14 days on sensitivity analysis using multiple imputation for missing data. Factors negatively associated with the primary outcome included a new diagnosis of diabetes (i.e., first time hyperglycemia presentation) and recent hospitalization within the past 30 days on sensitivity analysis only. The results of this study may aid ED physicians in making decisions for follow‐up and disposition for patients presenting for hyperglycemia in order to prevent unplanned recurrent visits and other adverse outcomes. While these factors require external validation, identifying individuals with these characteristics should be the focus of targeted clinical and educational interventions for future studies.

### 4.2 Previous Studies

Several retrospective studies have attempted to identify patients at higher risk of recurrent visits to the ED for hyperglycemia. A health records review performed by our study group at the same four academic tertiary care EDs found five risk factors and two protective factors for 30‐day recurrent hyperglycemia visits [5]. These risk factors included a previous hyperglycemia visit within the past month, age < 25 years, initial blood glucose < 20 mmol/L, having a family physician, and being on insulin. The protective factors included a systolic blood pressure between 90 and 150 mmHg and a heart rate > 110 beats per minute. Another health records review examining over 90,000 hospitalizations in the US‐based 2018 Nationwide Readmission Database found that having a Charlson Comorbidity Index of > 3, hypertension, female sex, and leaving against medical advice were predictors of all‐cause readmission following an initial hospitalization for diabetic ketoacidosis [12]. Recently, a large population‐based study in Ontario evaluated over 700,000 index ED visits for hyperglycemia over a 10‐year period and found that statistically significant predictors of a recurrent visit included: male sex, type 1 diabetes, regions with fewer visible minority groups and with less education or employment, higher hemoglobin A1C, more family physician or internist visits within the past year, being rostered to a family physician, previous ED visits in the past year, ED or hospitalization within the previous 14 days, access to homecare services, and previous hyperglycemia encounters in the past 5 years, while alcoholism and depression or anxiety were positive predictors for those aged 18–65 years [13]. Collectively, these studies have identified multiple potential predictors of adverse outcomes in this important patient population, but conclusions are unfortunately limited based on the potential biases introduced by the retrospective nature of their data collection.

Two recent systematic reviews have also examined predictors of 30‐day unplanned healthcare resource utilization in individuals with hyperglycemia or diabetes. A systematic review and meta‐analysis conducted by Soh et al. studied adults with diabetes and found that the strongest predictors of 30‐day unplanned hospital readmission for any reason were comorbidities, such as heart failure and renal disease, as well as insulin therapy and insurance status [6]. Additionally, a systematic review performed by Siddiqi et al. focused on ED utilization by describing variables associated with additional adverse outcomes including recurrent ED visits related to hyperglycemia and death, and not only hospitalization within 30 days [7]. Predictors of adverse outcomes in this review included age, lowest income quintile, urban dwellers, presence of comorbidities, coexisting hyperlactatemia, having a family physician, elevated serum creatinine level, diabetes managed with insulin, sentinel visit for hyperglycemia in the past month, and high blood glucose level measured in the ED. Almost all the included studies in both systematic reviews used retrospective data collection (i.e., they were health records reviews or case–control studies), and the only two studies that were prospective were primarily studying the effect of diabetes on patients with sepsis [14], and the characteristics of hyperglycemia in patients without a history of diabetes [15], thus not specifically examining patients with diabetes and their risk of recurrent healthcare resource utilization for hyperglycemia, which is the population of interest in our study.

It is noteworthy that having a history of substance use was the strongest independently associated risk factor for a recurrent ED visit for hyperglycemia within 30 days in our study. This variable included both patients who were actively using substances as well as those with a documented history of past substance use according to electronic medical records review, but not ascertained prospectively on telephone follow‐up with patients. The relationship between substance abuse and ED visits for diabetes and hyperglycemia has not been well elucidated in the past literature, although the previously mentioned population‐based study in Ontario described alcoholism and depression or anxiety as a risk factor for those aged 18–65 years [13]. We identified only one study by Hudon et al. that attempted to evaluate predictive factors of chronic frequent ED utilization specifically in individuals with diabetes [16]. In this study, 2.6% of patients with diabetes were identified as having chronic frequent ED utilization, defined as having ≥ 3 ED visits for three consecutive years, and the cumulative effect of a high illness burden (comorbidity index, chronic obstructive pulmonary disease, and injury) combined with mental health disorders (substance abuse and mental health disorders other than dementia) was associated with an increased risk of chronic frequent ED utilization. However, other studies have found a positive association between substance use and higher ED and healthcare resource utilization in general [17]. This is also true for adolescents and younger adults, as a study by Kim et al. found that polysubstance use (in particular, drugs considered “illicit” such as opioids, cocaine, and stimulant use) was strongly associated with repeated ED visits compared with the use of substances such as cannabis, alcohol and sedatives [18]. Future research should explore the influence of substance use in patients with diabetes presenting to the ED for hyperglycemia and how adverse outcomes may be reduced with interventions aimed at targeting treatment of both hyperglycemia and substance use disorders.

## 5 Limitations

Our study's strengths include its multicentre prospective design, including telephone follow‐up, permitting us to obtain data not routinely collected in health records reviews, including assessing for healthcare resource utilization at centres not directly linked to our hospital networks. However, our study had several limitations. We were only able to successfully reach 367 of 594 (61.8%) patients for telephone follow‐up, resulting in significant missing data for variables that were collected in this manner, namely characteristics such as race, education, and socioeconomic status or income data. We attempted to mitigate this limitation by performing a sensitivity analysis using multiple imputation, which showed similar results compared to our main regression. It is also possible that the outcome rates in our study may be an underestimate of the true incidence of recurrent ED visits for hyperglycemia at 30 days if patients presented to centres not linked with our hospitals' electronic health record or did not report this outcome on telephone follow‐up. This limitation was also mitigated by the fact that our two London sites' electronic health records are linked with the electronic records of 13 EDs in our region, and we could thus capture our hospital‐based outcomes, including our primary outcome, if patients presented to any of these surrounding hospitals. Additionally, the telephone follow‐up alone may have influenced patient behavior and decreased ED revisits (i.e., Hawthorne effect) [19], which may have contributed to the underestimation of our primary outcome. Furthermore, the reliance on telephone follow‐up could have led to response biases, as certain groups may have been less likely to participate, particularly if they were non‐English‐speaking or did not have access to a telephone, thus potentially leading to the under‐representation of racial and ethnic minorities or those of lower socioeconomic status. Finally, since our study was conducted at four urban academic tertiary care EDs in Ontario, our findings may not be generalizable to smaller, rural, or community hospital settings or to countries where there is not a public, universal healthcare system.

## 6 Conclusions

We conducted a multicentre prospective cohort study identifying predictors of 30‐day recurrent ED visits for hyperglycemia. We found that predictors of this outcome included having a history of substance abuse and initial laboratory blood glucose level, as well as having a recent ED visit within the past 14 days on sensitivity analysis. Factors negatively associated with a recurrent unplanned ED visit for hyperglycemia included a new diagnosis of diabetes and recent hospitalization within the past 30 days on sensitivity analysis only. Clinicians should be aware of these factors when disposition planning and arranging subsequent follow‐up for ED patients presenting for hyperglycemia. Future research will focus on studying interventions to mitigate the potentially modifiable risk factors to reduce adverse outcomes and reduce healthcare system costs for this important patient population.

## Author Contributions

J.W.Y. conceived the study and obtained research funding. J.W.Y. and K.V.A. designed the study. J.T. acquired and analyzed the data, while N.W. and I.K. conducted the regression analysis. All authors assisted with data interpretation. J.W.Y. drafted the manuscript, and all authors contributed substantially to its revision and also support its publication.

## Conflicts of Interest

The authors declare no conflicts of interest.

## Supporting information


**Appendix S1:** STROBE checklist.

**Data S1:** acem70133‐sup‐0002‐Supinfo.zip.

## Data Availability

The data that support the findings of this study are available from the corresponding author upon reasonable request.
