# Supplementary material for: Predictors of 30‐Day Recurrent Emergency Department Visits for Hyperglycemia in Patients With Diabetes: A Multicentre Prospective Cohort Study
Source: Acad Emerg Med. 2025 Aug 27;32(12):1309–19. doi: 10.1111/acem.70133 (PMC12690232; doi:10.1111/acem.70133)
Supplement: Supplementary file 2 — Data S1: acem70133‐sup‐0002‐Supinfo.zip. [file ACEM-32-1309-s001.zip › 10-14 day Telephone Follow-up -17 Jan 20- Clean copy.docx]

**Hyperglycemia Study**

**10-14 Day follow up data sheet**

Case #: ____________

RA: ______________ Date: _____________

**Attempts to contact by telephone:**⁬1⁬2⁬ 3

**Phone Script:**Ask to speak with participant.

“Hello, am I speaking to (participant’s name)? My name is ________ and I am a research assistant with the Department of Emergency Medicine at the London Health Sciences Centre. As you are aware, you were seen in the emergency department for high blood sugar. I’m calling you today to ask a short series of questions to determine how your health has been since you were discharged from the emergency.This should take less than five minutes of your time. Do you have time to discuss this right now?”

(If no: “When would be a more convenient time to call back?” ______________)

**Approximately how long have you known you have had diabetes or high blood sugar (years)? ________________**

**Is there a doctor that follows you regularly for your diabetes?** ⁬ Yes ⁬ No

**If yes, who?** ⁬Family doctor ⁬Internal medicine

⁬Endocrinologist/Diabetes Specialist ⁬Other: ___________________________

**Before coming to the emergency, when was the last time you saw this doctor for your diabetes?**

**___________________**

**After being discharged from this most recent visit to the emergency, did you take time off of work or school for this medical problem?**⁬ Yes ⁬ No NA **If yes, how many days?**_____________

**Did you see another doctor for this medical problem after you were discharged?**⁬ Yes ⁬ No

**If yes, who did you see?**

⁬Family doctor ⁬Another emergency doctor ⁬ Walk-in clinic ⁬Internal medicine

⁬Endocrinologist/Diabetes Specialist ⁬Other: ___________________________

**Did you have to adjust any of your diabetes medications after you were discharged?** ⁬ Yes ⁬ No

**If yes, what did you adjust?** Insulin: ⁬ Increased dose ⁬Decreased dose ⁬Started

Diabetes pills: ⁬ Increased dose ⁬Decreased dose ⁬Started Switched type

**Were you admitted to another hospital for this medical problem after you were discharged?** ⁬ Yes ⁬ No

**If yes, for how many days? ____________**

**The following questions may be more sensitive for some people to answer. Although we would be grateful if you could answer the next few questions, please let us know if you are not comfortable answering them:**

**What is the highest level of education you have completed?**

No schooling completed ⁬

Grade 8/Elementary graduate ⁬

High school diploma ⁬

College diploma ⁬

Bachelor’s degree ⁬

Masters, doctorate or professional ⁬

Prefer not to say ⁬

**What ethnic minority or racial heritage do you identify with?**

White or Caucasian ⁬

Native American/Aborginal ⁬

East Asian / Pacific Islander ⁬

South Asian (e.g. Indian, Pakistani, etc.) ⁬

Middle Eastern/Arab ⁬

Black or African Canadian ⁬

Hispanic or Latino ⁬

Other: ___________________ ⁬

Prefer not to say ⁬

**What is your household’s approximate yearly income?**

Less than $25,000 ⁬

$25,000 to $49,999 ⁬

$50,000 to $74,999 ⁬

$75,000 to $99,999 ⁬

$100,000 or more ⁬

Prefer not to say ⁬

**May we contact you in the future for other research opportunities regarding high blood sugar?**

Yes No

**Comments** ________________________________________________________________________________________________________________________________________________________________________________________________________________________________________________________________________________________________
