# Supplementary material for: Predictors of 30‐Day Recurrent Emergency Department Visits for Hyperglycemia in Patients With Diabetes: A Multicentre Prospective Cohort Study
Source: Acad Emerg Med. 2025 Aug 27;32(12):1309–19. doi: 10.1111/acem.70133 (PMC12690232; doi:10.1111/acem.70133)
Supplement: Supplementary file 2 — Data S1: acem70133‐sup‐0002‐Supinfo.zip. [file ACEM-32-1309-s001.zip › 30 day follow-up phone call 17 Jan 20- Clean copy .docx]

**Hyperglycemia Study**

**30 Day follow up data sheet**

Case #: ____________

RA: ______________ Date: _____________

**Attempts to contact by telephone:** ⁬1 ⁬2 ⁬ 3

**Phone Script:** Ask to speak with participant.

“Hello, am I speaking to (participant’s name)? My name is ________ and I am a research assistant with the Department of Emergency Medicine at the London Health Sciences Centre. As you are aware, you were seen in the emergency department for high blood sugar. I’m calling you today to determine how your health has been since you were discharged from the emergency. This should take less than two minutes of your time. Do you have time to discuss this right now?”

(If no: “When would be a more convenient time to call back?” ______________)

**Did you see a doctor for this medical problem after you were discharged?**⁬ Yes ⁬ No

**If yes, who did you see?**

⁬Family doctor ⁬Another emergency doctor ⁬ Walk-in clinic ⁬Internal medicine

⁬Endocrinologist/Diabetes Specialist ⁬Other: ___________________________

**Were you admitted to another hospital for this medical problem after you were discharged?** ⁬ Yes ⁬ No

**If yes, for how many days? ____________**

**Did you return to an external emergency room for High Blood Sugar after you were discharged?**  Yes ⁬ No

If yes, which hospital and how many visits? _________________________________________________

What was the final diagnosis and disposition? _____________________________________________________
